# Identification and Functional Characterization of the *Caenorhabditis elegans* Riboflavin Transporters *rft-1* and *rft-2*

**DOI:** 10.1371/journal.pone.0058190

**Published:** 2013-03-06

**Authors:** Arundhati Biswas, Daniel Elmatari, Jason Rothman, Craig W. LaMunyon, Hamid M. Said

**Affiliations:** 1 Departments of Medicine and Physiology/Biophysics, University of California Medical School, Irvine, California, United States of America; 2 Veterans Affairs Medical Center, Long Beach, California, United States of America; 3 Department of Biological Sciences, California State Polytechnic University, Pomona, California, United States of America; University of Pennsylvannia, United States of America

## Abstract

Two potential orthologs of the human riboflavin transporter 3 (hRFVT3) were identified in the *C. elegans* genome, Y47D7A.16 and Y47D7A.14, which share 33.7 and 30.5% identity, respectively, with hRFVT3. The genes are tandemly arranged, and we assign them the names *rft-1* (for Y47D7A.16) and *rft-2* (for Y47D7A.14). Functional characterization of the coding sequences in a heterologous expression system demonstrated that both were specific riboflavin transporters, although the *rft-1* encoded protein had greater transport activity. A more detailed examination of *rft-1* showed its transport of riboflavin to have an acidic pH dependence, saturability (apparent Km = 1.4±0.5 µM), inhibition by riboflavin analogues, and Na^+^ independence. The expression of *rft-1* mRNA was relatively higher in young larvae than in adults, and mRNA expression dropped in response to RF supplementation. Knocking down the two transporters individually via RNA interference resulted in a severe loss of fertility that was compounded in a double knockdown. Transcriptional fusions constructed with two fluorophores (*rft-1::GFP*, and *rft-2::mCherry*) indicated that *rft-1* is expressed in the intestine and a small subset of neuronal support cells along the entire length of the animal. Expression of *rft-2* is localized mainly to the intestine and pharynx. We also observed a drop in the expression of the two reporters in animals that were maintained in high riboflavin levels. These results report for the first time the identification of two riboflavin transporters in *C. elegans* and demonstrate their expression and importance to metabolic function in worms. Absence of transporter function renders worms sterile, making them useful in understanding human disease associated with mutations in hRFVT3.

## Introduction

Riboflavin, or vitamin B2, is critical to animal metabolism due to its role in the synthesis of flavin adenine dinucleotide (FAD) and flavin mononucleotide (FMN). These derivatives function as coenzymes that participate in a wide variety of the oxidation-reduction reactions of intermediate metabolism [1]. Humans with riboflavin deficiency suffer from numerous problems including neurological disorders and anemia [2]. Riboflavin also appears to reduce the risk for several cancers [3]–[6]. Human riboflavin uptake occurs via three transporters: hRFVT1, hRFVT3, and hRFVT2, although hRFVT3 appears to be primarily responsible for riboflavin uptake into the intestine [7]. Numerous mutations in hRFVT3 have been identified as the basis for the ALS-like disease Brown–Vialetto–Van Laere syndrome (BVVLS), which includes palsies, deafness, and respiratory problems [8]–[9]. BVVLS can be treated by riboflavin administration [10]. The mutations were shown to disrupt either subcellular targeting or activity of the transporter [11].

Like humans, the nematode *C. elegans* lacks the biosynthetic machinery necessary to synthesize riboflavin, being dependent solely upon dietary uptake. Many free-living nematodes, including *C. elegans,* consume bacteria, which typically produce abundant riboflavin [12]. Little work has been done on *C. elegans* vitamin transporters, although the mechanism of folate uptake has been shown to depend upon expression of the *folt-1* gene [13]–[14]. In one study demonstrating the importance of riboflavin to worms, riboflavin supplementation relieved lactic acidosis associated with mutations in genes encoding subunits of complex I of the mitochondrial respiratory chain [15]. Here, we describe two *C. elegans* riboflavin transporters, *rft-1* and *rft-2,* identified by a protein BLAST search using hRFVT3. We show that both *rft-1* and *rft-2* transport riboflavin and are expressed in the intestine.

## Methods

### Worm Husbandry

Most of the work was carried out using wild-type strain N2, although we also used *unc-119(ed3) III* worms for microinjection transformation. Worms were maintained under standard conditions and were fed *E. coli* strain OP50 [16]. For certain experiments, the NGM agar was supplemented with riboflavin, riboflavin analogues, or with other factors for growth of bacteria carrying RNAi feeding plamids. In egg laying assays, worms reared under experimental conditions were placed individually on small plates for approximately 2 hours (the exact time for each worm was recorded). Any eggs laid were counted and incubated at 20°C for 24 hours, at which point remaining unhatched eggs were considered to have failed to develop properly. The Caenorhabditis Genetics Center at the University of Minnesota supplied the worm strains.

### Cloning the Putative Riboflavin Transporters

The only two gene models in the *C. elegans* genome that bear significant homology to the hRFVT3 gene via BLASTp and tBLASTn searches were the tandemly arranged Y47D7A.16 and Y47D7A.14. We amplified the cDNAs of these two coding sequences from wild-type worms (strain N2). After removing the worms from plates, rinsing them four times in M9 buffer, and freezing them, we sonicated the worms in the presence of TRIzol® to extract total RNA (Life Technologies™, Rockville, MD). The RNA was treated with RQ1 DNase (Promega Corp., Madison, WI), extracted with an equal volume of phenol/choloroform/isoamyl alcohol (25∶24:1), precipitated with ethanol, and resuspended in nuclease-free H_2_0. cDNA was synthesized with the ProtoScript® AMV First Strand cDNA Synthesis Kit (New England Biolabs, Ipswich, MA). All reactions were carried out according to manufacturer’s protocols.

For Y47D7A.16, we initially attempted to amplify the cDNA with primers located just outside the predicted coding sequence but were unsuccessful by standard PCR. The primers were rft-1_outF (5′-TTTAGCAGTGTACCTAGGCAGT-3′) and rft-1_outR (5′-CGGGATTGATTGTGCAAAACCA-3′). However, rft-1_outF produced the correct sized product when combined with a reverse primer located in exon 3 (rft-1_exon3R: 5′-GGAGATTGCATCAACGATGG-3′). Further, a forward primer in exon 3 (rft-1_exon3F: 5′-ACTCCACATTATTATCCACC-3′) generated the expected product in combination with a reverse primer in exon 6 (rft-1_exon6R: 5′-GATACGATGCCAGTGGAGATATCAC-3′). Therefore, we knew that the 5′ end of the coding sequence was correct through part of exon 6. A different 3′ exonic structure was suggested by an EST sequence (WormBase ID: MM454_contig07814), and we designed another downstream primer to test this prediction (rft-1_alt-outR: 5′-AGTGAGACTAATGTGAAGTAAAGG-3′). Amplification with this new primer and rft-1_outF produced a product of the predicted size, thus confirming the new structure. We amplified the cDNA sequence for cloning using Expand High-Fidelity PLUS PCR system (Roche Diagnostics) and primers rft-1_XhoIF (5′-GAGA**CTCGAG**GTCATGAAAACGTTCCTGTTC-3′; XhoI site in bold and start codon underlined) and rft-1_NotIR (5′-TGTG**GCGGCCGC**TGTCACCGACATGATGGTGCAG-3′; NotI site in bold and complement of stop codon underlined). Following XhoI/NotI digestion, the PCR product was cloned into pCI-neo (Promega, Madison, WI), and sequencing showed the predicted exon structure was correct.

Cloning Y47D7A.14 was straightforward because the predicted gene model was correct. We again used Expand High Fidelity Plus polymerase and amplified the correct sized product with the primers rft-2_XhoIF (5′-CACA**CTCGAG**CAGGCAGAAATGGGTTGCTC-3′; XhoI site in bold and start codon underlined) and rft-2_NotIR (5′-TGTG**GCGGCCGC**TGCTAAGAGATTGATTTGCATT-3′; NotI site in bold and complement of stop codon underlined). The PCR product was cloned into pCI-neo, and sequencing showed the predicted exon structure was correct.

### Functional Expression of rft-1 and rft-2 in ARPE-19 Cells

ARPE-19 cells are derived from human retinal pigment epithelia and are characteristic of such cells *in vivo* [17]. These cells have been widely used to characterize the function of transporters including those of *C. elegans* [18]–[20]. We expressed the putative *rft-1* and *rft-2* in these cells using the Lipofectamine 2000 transfection system according to the manufacturer’s instruction (Invitrogen). ARPE-19 cells (60–70% confluent) were transiently transfected with 3 µg of *rft-1* and *rft-2* constructs per well of a 12-well plate. The cells were then incubated at 37°C for 48 hours and used for uptake studies. Cells transfected with vector alone (without DNA insert) served as controls (i. e., to determine endogenous riboflavin uptake activity). Uptake of [^3^H] riboflavin was determined at 37°C (5 min; initial rate) in Krebs-Ringer buffer (in mM: 133 NaCl, 4.93 KCl, 1.23 MgSO4, 0.85 CaCl2, 5 glucose, 5 glutamine, 10 HEPES, and 10 MES). The [^3^H]-radioactivity taken up by the cells was determined by means of scintillation counting and protein level (cell digest) was measured in parallel wells (Bio-Rad, Richmond, VA). Riboflavin uptake activity by the induced carrier was determined by subtracting uptake by cells transfected with empty vector from cells expressing *rft-1* and *rft-2*.

### Generation of RNAi Feeding Plasmids for rft-1 and rft-2

RNAi feeding plasmids were constructed by cloning portions of the cDNAs for *rft-1* and *rft-2* into the *C. elegans* RNAi expression vector pPD129.36 [21]. For *rft-1,* a 637 bp segment of the cDNA was amplified from RNA by RT-PCR using the primers rft-1_RNAiF (5′-TGTG**GCGGCCGC**TGGGGCTTAAGTGCTCTAATTCC-3′; NotI site in bold and gene specific underlined) and rft-1_RNAiR (5′-GAGA**CTCGAG**GGATAATAGTACCCGCAGTTTC-3′; XhoI site in bold and gene specific underlined). For *rft-2*, a 546 bp segment of the cDNA was amplified using the primers rft-2_RNAiF (5′-CACA**CTCGAG**TCCAGATCGCTTGTATAGTACC-3′; XhoI site in bold and gene specific underlined) and rft-2_RNAiR (5′-TCTC**GCGGCCGC**TCCAGATCCTTTTTCAGCGGAG-3′; NotI site in bold and gene specific underlined). The L4440 vector and the RT-PCR products that had been NotI/XhoI digested were ligated, and the ligations transformed into *E. coli* strain DH5α. Recombinant plasmids recovered by miniprep were subsequently transformed into *E. coli* strain HT115(DE3). In preparation for feeding assays, single colonies were grown overnight at 37°C in 25 ml LB broth containing 75 µg/ml carbenicillin and 12 µg/ml tetracycline. The next morning, 5 ml was transferred to 20 ml fresh media and grown to ∼0.4 OD_600_, when Isopropyl β-D-thiogalactoside (IPTG) was added to give a final concentration of 1.0 mM. After 1 hour at 37°C, the culture was used to seed NGM agar plates containing 6 mM IPTG and 25 µg/ml carbenicillin, and the plates were incubated overnight at room temperature.

### Quantitative PCR Determination of Transcript Abundance for rft-1

In order to understand the expression of *rft-1*, we reared worms under various conditions, extracted their total RNA, and performed quantitative RT-PCR. Worms were reared from egg on *E. coli* HT115(DE3) cells transformed with the RNAi feeding vector for *rft-1.* Other worms were reared from egg on HT115 cells transformed with the empty RNAi vector L4440, either on standard plates or plates supplemented with 2.5 mM riboflavin. After a given exposure interval to the RNAi bacteria, the worms were removed from the plates, rinsed four times in M9 buffer, and total RNA extracted using the TRIzol protocol described above. After DNase treatment, RNA was used as a template in reverse transcription (iScript cDNA synthesis kit; Bio-Rad), which was initiated using random oligonucleotides and carried out in a DNA thermal cycler (Light Cycler PCR System, Bio-Rad Laboratories, Hercules, CA) as per the manufacturer’s instructions. Reverse transcription was then followed by real-time PCR in 96 well plates with SYBR Green kit (Bio-Rad) with the gene-specific primers and the primers of β-actin (housekeeping gene). A 755 bp portion of the *rft-1* transcript was amplified with primers rft-1_Exon2F (5′-GGTGTTAGCATGTATTTGTC-3′) and rft-1_Exon6R2 (5′-GCGAAGAGCATAATGTTAGA-3′). As a control for total RNA in the samples, we amplified a 954 bp region of the β-actin homolog *act-2* with primers actinF (5′-GTATGGGACAGAAAGACTCG-3′) and actinR (5′-CGTCGTATTCTTGCTTGGAG-3′). Data were normalized relative to β-actin and then quantified as described by the vendor (Bio-Rad) [22].

### Construction and Analysis of Transcriptional Fusion Reporters for rft-1 and rft-2

The promoters of *rft-1* and *rft-2* were placed upstream of GFP and mCherry, respectively, utilizing the PCR fusion protocol described by Hobert [23]. Here, we used iProof™ High Fidelity DNA Polymerase (Biorad Laboratories, Richmond, CA) to combine 1,459 bp upstream of the *rft-1* start codon with GFP followed by the *unc-54* 3′ UTR from plasmid pPD95.75. For *rft-2*, we combined 2,647 bp upstream of the start codon with the mCherry coding sequence and the *unc-54* 3′ UTR from pCFJ104. Both constructs were purified with the Wizard PCR purification system from Promega (Madison, WI) and were co-injected into the gonads of *unc-119(ed3)III* worms. Included in the injection mix was the plasmid pCFJ151, which contains a wild type copy of the *unc-119* gene from *C. briggsae* (*Cbr-unc-119).* The *Cbr-unc-119* gene rescues the *unc-119(ed3)* mutation, so the transgenic worms crawled normally, which gave them a competitive advantage over the non-transgenic animals that arise every generation through loss of the extrachromosomal array of transgenes. In this way, transgenic animals are maintained over many generations without the need for active enrichment.

Worms that transmitted the transgenes stably from one generation to the next were analyzed microscopically on a Nikon Eclipse Ti inverted microscope outfitted for Nomarski DIC and epifluorescence. Worms at the L4 stage were anesthetized with 0.01 M sodium azide in M9 buffer [24], placed on slides and imaged for GFP and mCherry, and they were also imaged in the blue channel to obtain an image of the autofluorescence of the intestine for use as an internal control. Using Nikon NIS Elements software, an analysis line was drawn down the length of the intestine, halfway between the lumen and the edge of the intestine. The average intensity of the fluorescence along the line was calculated for the three channels. The GFP and mCherry fluorescence for each worm were calculated as the ratio of the blue channel, thus correcting for intestine autoflorescence in the green and red channels, slight differences in worm age, and placement of the analysis line. We analyzed 44 worms reared on standard NGM agar and 46 worms reared on agar supplemented with 2.5 mM riboflavin.

### Statistical Analysis

Uptake data with ARPE19 cells transfected with *rft-1* and *rft-2* are presented as the means ±SE of at least three separate experiments expressed in femtomoles per milligram of protein per unit of time. Univariate ANOVA was used to determine statistical significance among means, and the Bonferroni post-hoc test was used in comparing pairs of means. *P*<0.05 was considered statistically significant. Induced carrier-mediated riboflavin uptake was determined by subtracting uptake by endogenous from total uptake. Other determinations were run on at least three different occasions using three different preparations.

## Results

### Cloning of the Riboflavin Transporters from C. Elegans

**Figure 1:**
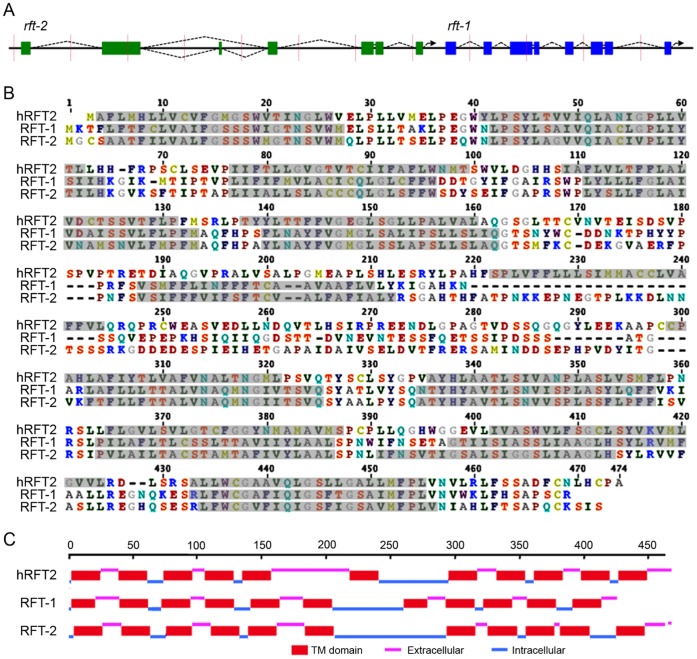
Exonic and protein structure of riboflavin transporters. (A) The tandem arrangement of *rft-1* and *rft-2* on chromosome V. Note that *rft-2* is alternatively spliced. Hash marks along the chromosome indicate 1,000 bp intervals. (B) Amino acid alignment of *rft*-1, *rft*-2, and the putative human homolog, hRFVT3. Shaded regions indicate predicted transmembrane domains. (C) The locations of the transmembrane domains and intracellular and extracellular loops. The numbered bar indicates amino acid position.

A search of the *C. elegans* genome for potential homologs of the human riboflavin vitamin transporter 3 (hRFVT3; Accession NP_212134) identified two predicted genes. The genes reside in tandem on Chromosome V (Fig. 1a): Y47D7A.16 is 33.7% identical to hRFVT3, and Y47D7A.14 is 30.5% identical to hRFVT3. The two *C. elegans* putative paralogs are 60.4% identical. Based on riboflavin uptake results described below, we assign the name *rft-1* to Y47D7A.16 and *rft-2* to Y47D7A.14, where *rft* denotes riboflavin transporter. Cloning of the cDNA was problematic for *rft-1* due to a mistaken prediction of the 3′ end of the coding sequence. Based upon EST sequences, we identified the true 3′ end and cloned the entire 1,284 nucleotide cDNA sequence from poly(A)^+^ RNA by RT-PCR. Cloning *rft-2* was straightforward, as the predicted ends of the sequence were correct. However, this sequence is alternatively spliced to form two isoforms: *rft-2b* contains a third exon comprising 39 nucleotides missing in *rft-2a*. The shorter 1,392 base *rft-2a* is more common; it was present in 88% of the cloned RT-PCR products we sequenced. The RFT-1 and RFT-2a *C. elegans* riboflavin transporters are similar in length and transmembrane (TM) domain structure compared to hRFVT3 (Fig. 1 B and C). All three have 11 predicted TM domains with an extensive intracellular loop between the sixth and seventh TM domains. The RFT-2b isoform contains an additional 13 amino acids in the long intracellular loop.

### Functional Characterization of RFT-1 and RFT-2

**Figure 2:**
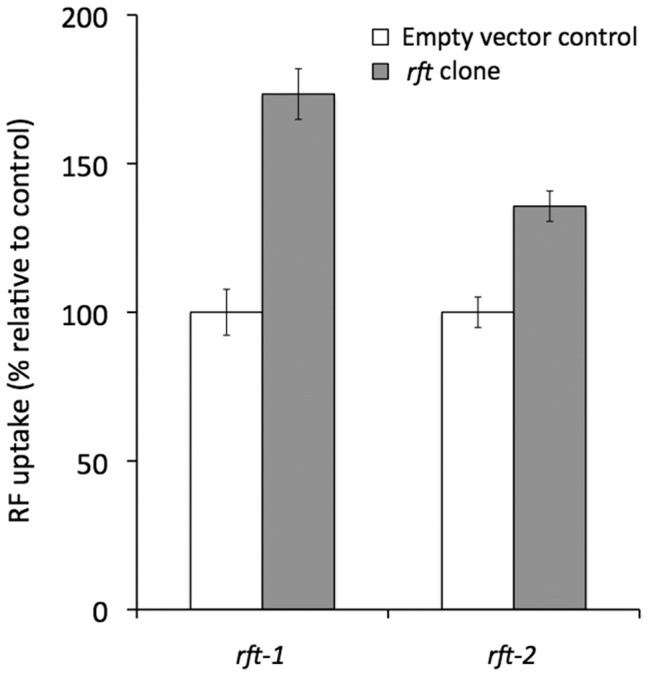
Functional identification of the cloned *rft*-1 and *rft*-2 as a specific riboflavin transporter in ARPE-19 cells. Confluent ARPE-19 cells expressing *rft-1 and rft-2* were incubated with [^3^H] riboflavin (14 nM; 5 min at 37°C in Krebs-Ringer buffer, pH 5.5). Carrier mediated uptake was determined as described in the methods section. Induced uptake by the riboflavin transporter was determined by subtracting uptake in empty-vector transfected cells from the cells transfected with the *rft*-1 and *rft*-2 constructs and are expressed as means ± SE.

**Figure 3:**
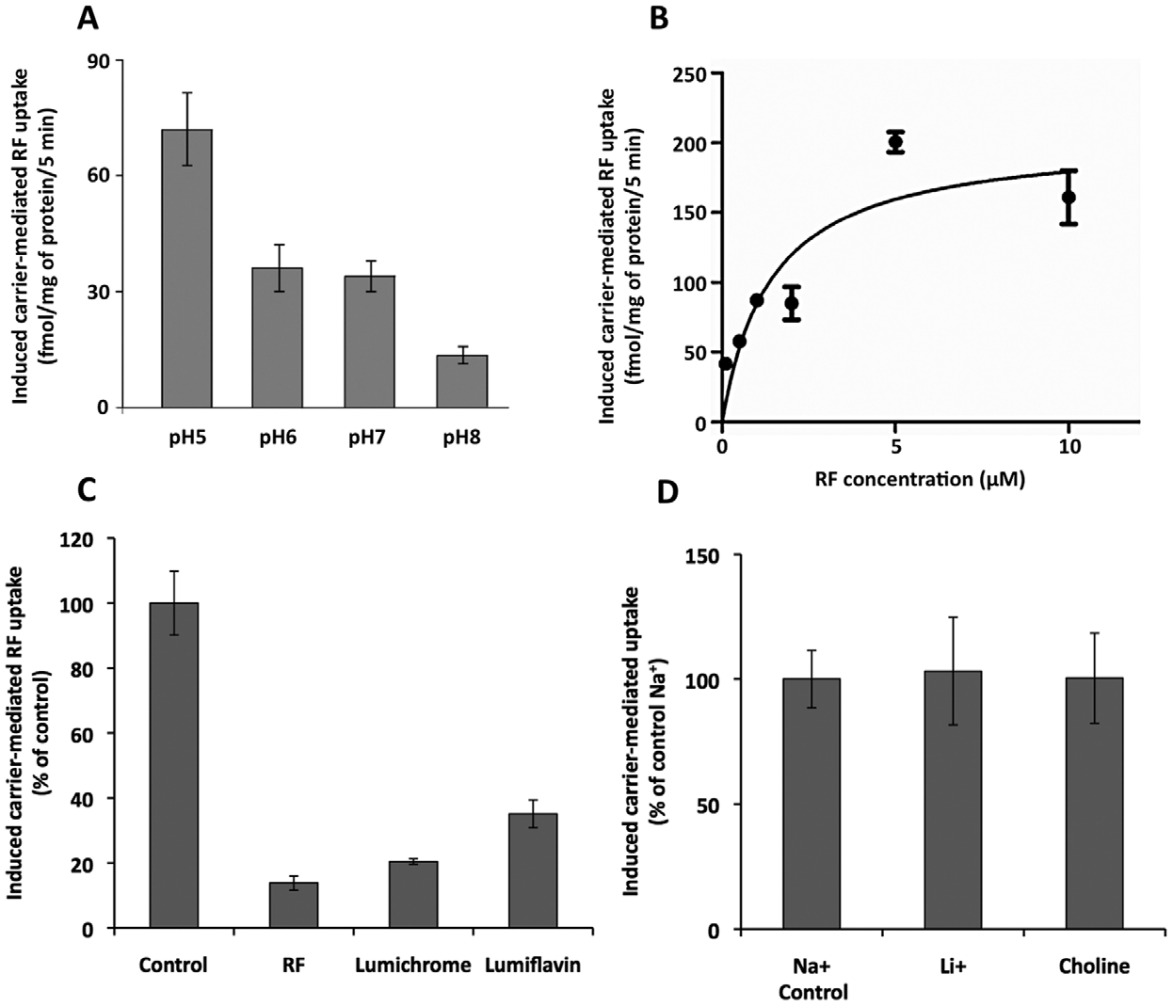
Characteristics of riboflavin uptake by ARPE-19 cells expressing *rft-1.* Data are means ± SE of at least 3 separate uptake determinations, and uptake by the induced carrier was determined as determined in Fig. 2. (A) Effect of incubation buffer pH on the induced [^3^H]-riboflavin uptake. (B) Initial rate of [^3^H]-riboflavin uptake as a function of concentration by the induced riboflavin carrier. (C) Effect of unlabeled riboflavin and its structural analogues on [^3^H]-riboflavin uptake by the induced carrier compared to control. (D) Role of Na^+^ in [^3^H]-riboflavin uptake by the induced carrier; Na^+^ was replaced with either Li^+^ or choline.

The cDNAs of *rft-1 and rft-2* were cloned into pCI-neo (Promega, Madison, WI) and transfected into ARPE-19 cells. Uptake of [^3^H]- riboflavin was assayed at 48 hours after transfection. Over the course of four trials, the rate of uptake for [^3^H] riboflavin was significantly higher for *rft-1* than for control cells containing empty vector alone, (Fig. 2; *F_1,25_* = 40.69, *P*<0.001). In a separate experiment with three trials, *rft-2* transfected cells also had significantly higher riboflavin uptake compared to controls (Fig. 2;; *F_1,21_* = 24.31, *P*<0.001). Given the close similarity of *rft-1* and *rft-2,* the fact that both transport riboflavin, and that the *rft-1* transporter has greater activity in our heterologous expression system, we focused our attention on *rft-1* for more extensive physiological characterization (effect of pH, kinetic parameters, effect of riboflavin analogs and role of Na^+^). The pH of the incubation buffer had a significant on riboflavin uptake (*F_3,11_* = 5.48, *P* = 0.024) with the greatest uptake at pH 5 (Fig. 3A). We also examined riboflavin uptake by the induced carrier in ARPE19 cells as a function of substrate concentration (0.01–10 µM) and observed clear saturation in uptake (Fig. 3B). The apparent *K*_m_ and *V*_max_ of the induced saturable uptake component were calculated by Graph Pad Prism and found to be 1.4±0.5 µM and 204.8±23.9 pmol/mg protein/5 min, respectively. Competition by unlabeled riboflavin and the structural analogues lumichrome and lumiflavin significantly inhibited [^3^H] riboflavin uptake in our heterologous expression system (Fig. 3C *; F_3,11_* = 51.70, *P*<0.001). We further tested the role of Na^+^ in the induced riboflavin uptake by *rft-1* expressing ARPE19 cells. Replacement of Na^+^ with equimolar concentrations of Li^+^ and choline had no effect on [^3^H] riboflavin uptake (Fig. 3D; *F_2,8_* = 0.11, *P* = 0.988). Given that Na^+^ appears unimportant for driving riboflavin uptake, our results showing increased uptake in lower pH suggests that transport is proton driven.

### Expression of rft-1

**Figure 4:**
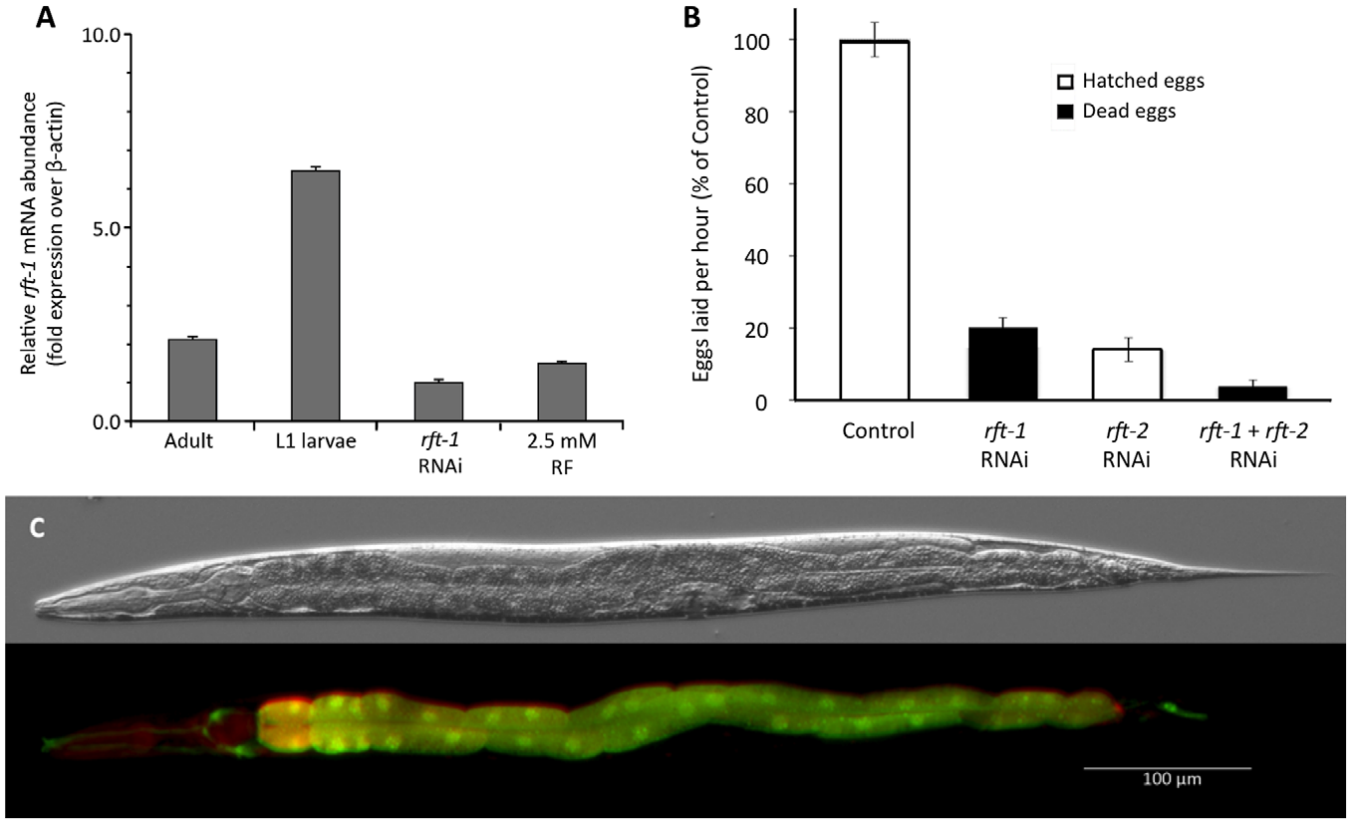
Expression and phenotypes of the riboflavin transporters. (A) Quantitative RT-PCR analysis of *rft-1* expression. The data were normalized relative to the housekeeping gene β-actin (*act-2)*. All worms were raised on HT115(DE3) bacteria harboring either the *rft-1* RNAi feeding plasmid (*rft-1* RNAi worms) or the empty vector, pPD129.36 (all other worms). Additionally, some worms were raised on plates supplemented with riboflavin. (B) The effects of RNAi on egg laying. Here, worms were reared on bacteria harboring the RNAi empty vector (Controls), the *rft-1* RNAi feeding vector, the *rft-2* RNAi feeding vector, or both RNAi feeding vectors. (C) Micrographs of a worm expressing the transcriptional reporters P*rft-1::gfp* and P*rft-2::mCherry*. Both reporters were expressed in the intestine, whereas only mCherry was found in the pharynx, and only GFP was found in neuronal support cells that spanned the length of the body.

Expression of *rft-1* mRNA was assessed via quantitative PCR. We found significant differences among all treatments (Fig. 4A; *F_3,11_* = 269.7, *P*<0.01, all treatment comparisons were different by Bonferroni post hoc testing at *P* = 0.001). The *rft-1* mRNA was at its highest expression relative to β-actin in L1 larvae and dropped significantly in adult worms (Fig. 4A). Exposure of the whole worm to *rft-1 *RNAi reduced mRNA abundance significantly, and supplementation of the culture media with a high exogenous concentration riboflavin also reduced transcript abundance (Fig. 4A). These results demonstrate that *rft-1* expression is regulated during development and that RNAi effectively down regulates transcript abundance. Further, *rft-1* expression is sensitive to systemic riboflavin and is expressed at lower levels when riboflavin is abundant.

Knockdown of both genes by RNAi resulted in reduced fertility in worms. Worms were fed bacteria engineered to express dsRNA corresponding to the mRNA of each gene. After being reared from egg on *rft-1, rft-2,* or a combination of *rft-1* and *rft-2* dsRNA expressing bacteria, worms experienced a severe reduction in the number of eggs laid (Fig. 4B; *F_3,89_* = 121.98, *P*<0.001). The response to *rft-1 *RNAi was more severe because none of the small number of eggs laid hatched (Fig. 4B). In an additional study, supplementation with 2.5 mM riboflavin in the agar partially rescued worms raised on a combination of *rft-1* and *rft-2* RNAi. Compared to the egg laying rate of worms raised on the combination of *rft-1* and *rft-2* RNAi alone (3.9% of controls; SEM = 1.8), riboflavin supplementation significantly increased egg laying rate to 23.6±5.5% of controls (*t* = −3.405; *P* = 0.005; t-test for unequal variances). Further, all the eggs on riboflavin supplementation hatched. Thus, a high concentration of exogenous riboflavin moderately relieved the congenital riboflavin deficiency induced by RNAi knockdown of riboflavin transport.

Both transporters are expressed in the intestine. We constructed transcriptional fusions for both genes, placing a different reporter coding sequence under control of the promoter of each gene. The *rft-1::GFP* transcriptional fusion showed strong expression throughout the length of the intestine (Fig. 4C). Interestingly, GFP labeling also occurred in what appear to be neuronal support cells that run the length of the body (Chris Link, personal communication). We have yet to determine the exact cells that are labeled. The transcriptional fusion *rft-2::mCherry* was expressed throughout the intestine and in the pharynx (Fig. 4C). The limitation of transcriptional fusions is that they do not indicate subcellular localization, but they do show the cells and tissues where expression occurs. We quantified the intensity of the fluorescence of the two reporters. When raised on agar containing 2.5 mM riboflavin, GFP fluorescence dropped to 81% of what it was on standard agar, and mCherry fluorescence dropped to 87% (riboflavin agar, N = 46, standard agar, N = 44; Effect of riboflavin supplementation: F_1,90_ = 3.873, P = 0.05) Thus, increased dietary riboflavin resulted in a significant drop in the expression of both reporters. Given the close proximity of (i) the *rft-1* start codon to the *rft-2* stop codon (Fig. 1; ∼400 bp), and (ii) the *rft-2* start codon to the stop codon of the upstream gene (*gnrr-5;* ∼700 bp), there is the possibility that these three genes form an operon [25]. Although there is no current evidence for such an arrangement, if it were true, the expression of all three genes would be driven by the promoter of the first gene in the sequence, *gnrr-5.* Our reporter expression patterns for *rft-1* and *rt-2* driven by the DNA flanking each gene is consistent with dietary riboflavin uptake and supports the hypothesis that these genes are expressed individually and not as part of an operon.

## Discussion

As with most animals, *C. elegans* worms must accrue riboflavin from dietary sources [26]. Uptake requires specific transporters in the intestine where riboflavin is released from ingested food - in the case of *C. elegans,* ingested bacteria. We have identified two transporters, *rft-1* and *rft-2*, that are potential homologs of hRFVT3. When cloned into a mammalian expression vector and expressed in a human cell line, both gene products resulted in riboflavin uptake. Further investigation of *rft*-1 showed that transport was blocked by riboflavin analogues, was Na^+^ independent, and was saturable [1.4±0.5 µM]. Like hRFVT3 [27], the *C. elegans rft*-1-mediated transport was most active in an acidic environment, which is reasonable given that the *C. elegans* intestine is likely to have an acidic pH [28].

The expression of *rft-1* is sensitive to the riboflavin status of the worm. Transcript abundance dropped when worms were exposed to exogenous supplement of riboflavin suggesting adaptive regulation by substrate level. Similarly, riboflavin transporter mRNA expression in rats (rRFVT3) and mouse pancreatic β-TC-6 cells (via the mouse RFVT-1) was adaptively regulated by riboflavin availability [27] [29]. Further, expression of our transcriptional GFP reporter driven by the *rft-1* promoter was down-regulated under riboflavin supplementation. While *rft-1* was expressed in the intestine, it was also found in neuronal support cells which function to protect neurons. It is unclear what function the transporter plays in these cells, although it is tempting to hypothesize a chemosensory function, given that substances may be sensed by the neuron after transport through its sheath cell [30]. Indeed, bacteria typically produce riboflavin [12], so an ability to sense a bacterial food source via riboflavin would be an adaptive feature. Of course, there is always the possibility that GFP fusions do not localize properly, so additional experimentation is necessary before this issue is resolved.

The expression of *rft-2* is different from that of *rft-1*. We found *rft-2* was uniquely expressed in the pharynx, and like *rft-1*, it was expressed along the entire length of the intestine. It is unclear how the different locations of expression relate to transporter function. In humans, three transporters are present in the intestinal epithelia: hRFVT1 is expressed mainly at the basolateral membrane, while hRFVT2 is localized to the apical membrane, with hRFVT3 (and hRFVT1) colocalizing to vesicles that are most likely endosomes [7]. Future studies are required to determine the subcellular localization of the two *C. elegans* riboflavin transporters.

What is clear is that like other animals, *C. elegans* worms take up riboflavin from dietary sources. The *rft-1* and *rft-2* encoded transporters appear to vary in function, each having a unique, but overlapping, set of tissues where expression occurs. Riboflavin is critical to metabolic function, and when these transporters fail to function, worms experience a riboflavin deficiency that renders them sterile. Humans who inherit mutations in hRFVT3 also suffer a debilitating disease, BVVLS [8], which responds to riboflavin administration [10]. In future studies we hope to determine the specific functions of the two *C. elegans* riboflavin transporters.
